# Account of Diseases in an Eastern District of London, from August 20, to September 20, 1803

**Published:** 1803-10-01

**Authors:** 


### *Account of Diseases in an Eastern District of London, from August 20, to September 20, 1803.*

| | |
|---|---|
| **ACUTE DISEASES.** | |
| Typhus | 6 |
| Scarlatina | 9 |
| Cholera | 7 |
| Dysenteria | 10 |
| Rheumatismus Acutus | 4 |
| **CHRONIC DISEASES.** | |
| Tussis | 10 |
| Dyspnœa | 15 |
| Tussis cum Dyspnœa | 7 |
| Hæmoptysis | 3 |
| Diarrhœa | 18 |
| Enterodynia | 6 |
| Ascites | 4 |
| Anasarca | 8 |
| Hæmorrhois | 6 |
| Prolapsus Ani | 4 |
| Leucorrhœa | 7 |
| Amenorrhœa | 12 |
| Menorrhagia | 5 |
| Prolapsus Vaginæ | 2 |
| Paralysis | 3 |
| Vertigo | 4 |
| Hypochondriasis | 4 |
| Rheumatismus Chronicus | 14 |
| **PUERPERAL DISEASES.** | |
| Enteritis | 2 |
| Dolores Post Partum | 7 |
| Œdema Puerperalis | 1 |
| **INFANTILE DISEASES.** | |
| Diarrhœa | 15 |
| Hydrocephalus Internus | 1 |
| Aphthæ | 7 |

The diseases which are most frequently observed in the autumnal months now begin to shew themselves. Disorders of the bowels are becoming very frequent, and, in some instances, prove very tedious and obstinate. Diarrhœa, Dysenteria, and Cholera are very general. The first of these, when restrained within certain limits, hardly deserves the name of disease, in any other sense than every effort of nature to throw off what is burdensome and offensive, when too violent or irregular, may be termed morbid. If a diarrhœa be not accompanied with pain, or if the pain be relieved by the intestinal discharge; if the appetite be not impaired, nor the strength of the patient too much reduced, this evacuation may be suffered to continue at least for some time, without any material injury. If however by the long continuance, or the excessive degree of it, the strength is much reduced; if the uneasiness and pain of of the bowels, which at first preceded the discharge, have ceased, and the evacuation seems to be continued either from weakness or habit, small doses of the pulv. rhei combined with the pulv. e cret comp. may be taken with advantage; but the too early application of astringent remedies is often attended with inconvenience, and sometimes with danger.

